# Immunological and tumor-intrinsic mechanisms mediate the synergistic growth suppression of experimental glioblastoma by radiotherapy and MET inhibition

**DOI:** 10.1186/s40478-023-01527-8

**Published:** 2023-03-13

**Authors:** Manuela Silginer, Eleanna Papa, Emese Szabó, Flavio Vasella, Martin Pruschy, Christopher Stroh, Patrick Roth, Tobias Weiss, Michael Weller

**Affiliations:** 1 Laboratory of Molecular Neuro-Oncology, Department of Neurology, University Hospital of Zurich, Frauenklinikstrasse 26, 8091 Zurich, Switzerland; 2 Laboratory of Molecular Neuro-Oncology, Department of Neurology, University of Zurich, Zurich, Switzerland; 3 Laboratory for Molecular Radiobiology, Department of Radiation Oncology, University Hospital and University of Zurich, Zurich, Switzerland; 4 Merck Healthcare KGaA, Darmstadt, Germany

**Keywords:** Glioma, HGF, Immunotherapy, Irradiation, Synergy

## Abstract

The hepatocyte growth factor (HGF)/MET signaling pathway has been proposed to be involved in the resistance to radiotherapy of glioblastoma via proinvasive and DNA damage response pathways.

Here we assessed the role of the MET pathway in the response to radiotherapy in vitro and in vivo in syngeneic mouse glioma models. We find that the murine glioma cell lines GL-261, SMA-497, SMA-540 and SMA-560 express HGF and its receptor MET and respond to exogenous HGF with MET phosphorylation. Glioma cell viability or proliferation are unaffected by genetic or pharmacological MET inhibition using tepotinib or CRISPR/Cas9-engineered *Met* gene knockout and MET inhibition fails to sensitize glioma cells to irradiation in vitro. In contrast, the combination of tepotinib with radiotherapy prolongs survival of orthotopic SMA-560 or GL-261 glioma-bearing mice compared with radiotherapy or tepotinib treatment alone. Synergy is lost when such experiments are conducted in immunodeficient *Rag1*^*−/−*^ mice, and, importantly, also when *Met* gene expression is disrupted in the tumor cells. Combination therapy suppresses a set of pro-inflammatory mediators including matrix metalloproteases that are upregulated by radiotherapy alone and that have been linked to poor outcome in glioblastoma. Several of these mediators are positively regulated by transforming growth factor (TGF)-β, and pSMAD2 levels as a surrogate marker of TGF-β pathway activity are suppressed by combination treatment. We conclude that synergistic suppression of experimental syngeneic glioma growth by irradiation and MET inhibition requires MET expression in the tumor as well as an intact immune system. Clinical evaluation of this combined strategy in newly diagnosed glioblastoma is warranted.

**Supplementary Information:**

The online version contains supplementary material available at 10.1186/s40478-023-01527-8.

## Introduction

Glioblastoma is an intrinsic malignant brain tumor probably derived from neuroglial progenitor cells characterized morphologically by invasive growth, focal necrosis and angiogenesis and on the molecular level by frequent genetic alterations involving the receptor tyrosine kinase (RTK)/RAS/phosphatidylinositol 3-kinase (PI3K), p53 and retinoblastoma (RB) pathways [50]. The standard of care for glioblastoma includes surgical resection as feasible followed by radiotherapy plus concomitant and maintenance temozolomide chemotherapy [47]. Several approaches of improving outcome by targeting angiogenesis have either entirely failed, e.g., cilengtide to target integrins [42], or resulted in prolonged progression-free, but not overall survival, e.g., bevacizumab to target vascular endothelial growth factor (VEGF) [8, 19]. Escape from anti-angiogenic therapy has been attributed to up-regulation of pro-invasive pathways and VEGF-independent pathways of angiogenesis involving, e.g., placental-derived growth factor (PlGF) [44], transforming growth factor-β [30] or hepatocyte growth factor (HGF) and its receptor MET [28]. MET expression has in fact been proposed as a marker for stemness in glioblastoma [23, 26, 34].

There is also increasing interest in the role of the HGF/MET pathway in the response of glioblastoma to radiotherapy. A radiosensitizing effect of MET inhibition may be partially mediated through inhibition of DNA double strand repair [48]. Accordingly, targeting MET by gene silencing potentiated responses to irradiation in the human U87MG [24] and U251 [9] intracranial glioma xenograft models. MET may protect from irradiation through increased *Met* mRNA expression in response to irradiation via activation of ataxia-telangiectasia mutated (ATM)–NF-kB signaling, a central mediator of the radiation-induced DNA damage response [14, 15].

HGF has also been implicated in the regulation of immune responses in different tumor models [20]. In the central nervous system, HGF protects from inadvertent immune responses by inducing tolerogenic dendritic cells and regulatory T cells [4]. A modulation of the tumor immune microenvironment in response to radiotherapy without or with MET inhibition in glioblastoma has not been studied. Here we explored the therapeutic efficacy and potential modes of action of MET-targeted therapy combined with irradiation in syngeneic murine glioma models in vitro and in vivo.

## Materials and methods

### Reagents and cell lines

EMD 1,214,063 (3-(1-(3-(5-(1-methylpiperidin-4-ylmethoxy)-pyrimidin-2-yl)-benzyl)-1,6-dihydro-6-oxo-pyridazin-3-yl)-benzonitrile) (tepotinib) is a highly specific, reversible and ATP-competitive, small molecule MET receptor tyrosine kinase inhibitor (Merck Healthcare KgaA, Darmstadt, Germany) with brain pentetration [5, 18, 25]. The drug was dissolved in DMSO and further diluted in cell culture medium. For in vivo studies, tepotinib was prepared with 20% solutol and 80% sodium acetate buffer (pH 5.5). Recombinant human HGF (GeneTex, Lucerne, Switzerland) was dissolved in Dulbecco's phosphate-buffered saline (PBS) and used at a 50 ng/ml. The murine glioma cell lines SMA-497, SMA-540 and SMA-560 were obtained from Dr. D. Bigner (Duke University Medical Center, Durham, NC) and GL-261 from the National Cancer Institute (Frederick, MD). The cells were cultured in Dulbecco's Modified Eagle Medium (DMEM) with 10% fetal calf serum (FCS) and 1% glutamine. Cells were regularly authenticated by short tandem repeat analysis (Microsynth AG, Balgach, Switherland), lastly in 2020, and tested for mycoplasms. Gene expression profiles for these cell lines have been generated previously [1].

### Real-time polymerase chain reaction (RT-PCR)

cDNA generated by reverse transcription of 1 µg total RNA was subjected to RT-PCR. Relative gene expression was measured as described [34] using a variation of the 2^(- delta delta CT) method [27]. Hypoxanthine–guanine phosphoribosyltransferase 1 (*Hprt1*) served as a reference gene. Mouse specific primer sequences were as follows: *Hprt1* fw: 5'- CCT AAG ATG AGC GCA AGT TGA A -3', rev: 5'- CCA CAG GAC TAG AAC ACC TGC TAA-3'; *Hgf* fw: 5'- TCA AAA TGT CAC CTA AAA CAA TCC -3', rev: 5'- ACA AAC AAT ACA ACA GAA AAC ACC -3'; *Met* fw: 5'- TTT GGG GAA GTC TCA TTT TTG -3', rev: 5'- CGA TTT TCA GTT GGC TTT TG -3'; *TGF-β*_*1*_ fw: 5'- TGG AGC AAC ATG TGG AAC TC -3', rev: 5'- GTC AGC AGC CGG TTA CCA -3'; *TGF-β*_*2*_ fw: 5'- GCC CAC TTT CTA CAG ACC CT -3', rev: 5'- CCT TGC TAT CGA TGT AGC GC -3', *TGF-β*_*3*_ fw: 5'- AGC ATC CAC TGT CCA TGT CA -3', rev: 5'- TTC TTC CTC TGA CTG CCC TG -3', *PAI-1* fw: 5'- TCT GGG AAA GGG TTC ACT TTA CC -3', rev: 5'- GAC ACG CCA TAG GGA GAG AAG -3', *PDGF-B* fw: 5'- TCC GTA GAT GAA GAT GGG GCT -3', rev: 5'- CGT CTT GCA CTC GGC GAT TA -3'.

### Immunoblot analysis

Lysates of cells or tumor specimens were prepared using radio-immunoprecipitation assay (RIPA) lysis buffer (pH 7.8) containing 25 mM Tris–HCl, 120 mM NaCl, 5 mM EDTA and 0.5% NP-40 supplemented with phenylmethylsulfonyl fluoride (Sigma Aldrich, St. Louis, MO), 200 mM sodium orthovanadate, 0.5 M sodium fluoride, protease inhibitor cocktail and phosphatase inhibitor cocktails 2 and 3 (Sigma Aldrich). Primary antibodies were as follows: rabbit anti-p-MET (Tyr1234/1235) (D26), mouse anti-MET (clone 25H2), rabbit anti-p-SMAD2 (Ser465/467) (138D4, all from Cell Signaling Technology, Denvers, MA) or goat anti-actin (sc-47778; Santa Cruz Biotechnology, Santa Cruz, CA). The membranes were exposed to horseradish peroxidase-conjugated secondary species-specific antibodies.

### Proteome profiler array

For the simultaneous determination of relative levels of mouse cytokines, the Mouse XL Cytokine Array Kit from R&D Systems was used (ARY028, Minneapolis, MN). Tissue lysates were prepared by electric homogenizer in ice-cold lysis buffer and supplemented with protease inhibitor cocktail, followed by centrifugation to remove cellular debris; 150 µg protein was used for each tumor sample and nitrocellulose membrane (control, monotherapies, combination therapy). To compare the proportion of protein levels in each tumor specimen, the quantification of pixel density of each spot of the array was performed using Image J (version 1.32j) software (National Institutes of Health, Bethesda, MD, http://rsb.info.nih.gov/ij/).

### Enzyme-linked immunosorbent assays (ELISA)

Mouse HGF was quantified with the Mouse HGF ELISA Kit (#EMHGF, Thermo Scientific, Frederick, MD) using serum-free supernatants of 4 × 10^6^ cells collected after 36 h.

### Invasion assays

Glioma cell invasiveness across Matrigel membranes was assessed using Corning BioCoat™ Matrigel Invasion Chambers (#354,480, 24-well format, pore size: 8 μm, Corning Life Sciences, Tewksbury, MA) and, for SMA-540 and SMA-560 only, across collagen type I by three-dimensional tumor spheroid invasion assay (96-well format). For matrigel invasion assays, serum-free medium supplemented with 50 ng/ml murine HGF was applied to the bottom well as chemoattractant. Serum-free medium alone served as negative control. After 22 h at 37 °C and 5% CO_2_, the invading cells on the bottom surface of the membranes were stained with Mayer's haematoxylin. Three microscopic fields per sample were analyzed at 10 × magnification. The spheroid invasion assay has been described [44].

### CRISPR/Cas 9 knockout of MET

Knockout clones were generated as described [29, 36]. Briefly, two *MET*-specific guide RNAs (5’-GAGAGCACGACAAATACGTA-3’ and 5’-GTATCGGACAGAGTTTACCA-3’) were cloned into pSpCas9(BB)-2A-GFP and lipofectamine (Thermo) was used to co-transfect both plasmids. pSpCas9(BB)-2A-GFP (PX458) was a gift from Feng Zhang (Addgene plasmid # 48,138). After puromycin selection, single cells were sorted into 96 well plates using a BD FACSAria III. Knockout was confirmed with RT-PCR and Sanger sequencing after expansion of clones.

### Cell doubling time

Cell doubling times were calculated during the exponential growth phase of cells based on the following formula: $\mathrm{doubling time}=\frac{\left(\mathrm{t}-{\mathrm{t}}_{0}\right) \times {\mathrm{log}}_{2}}{\mathrm{log}(\mathrm{n}-{\mathrm{n}}_{0})}$ (t, culture time; n, number of viable cells).

### Clonogenic survival assay

The cells were pre-treated as indicated and then seeded at low densitiy (100 cells per well) in 96-well plates, followed by observation for 7–14 days. Metabolic activity as a surrogate marker of viability was assessed using 3-(4,5-dimethylthiazol-2-yl)-2,5-diphenyltetrazolium bromide (MTT, Sigma-Aldrich).

### Animal studies

All experiments were conducted in accordance with the Swiss Cantonal Veterinary Office and according to guidelines of the Swiss federal law on animal protection and have been approved by the cantonal veterinary office. mRNA was prepared from untreated gliomas that started to render mice symptomatic or from non-tumor-bearing mouse brain. For treatment studies, 5,000 SMA-560 or 20,000 GL-261 tumor cells were stereotactically implanted into the right striatum of six to 12-week-old immunocompetent VM/Dk mice, immunocompetent C57BL/6 mice or immunodeficient Rag1^−/−^ (B6.129S7-Rag1tm1Mom/J) mice, devoid of mature B and T cells, on a C57BL/6 background. Typical experiments included 10 animals per group, 7 mice for survival analysis and 3 prerandomized mice for histological assessments at an early timepoint when the first mouse of any treatment group become symptomatic. For randomization of the groups, a free randomization software (www.randomizer.org) was used. When animals of both sexes and/or of different age were used, we created uniform blocks and randomized animals of each block to the different treatments groups of an experiment to balance for eventual sex- or age effects. Drug treatments and the analysis of the mouse experiments were done by different researchers. Tepotinib was administered orally using gavage at 100 mg/kg five days per week. Local cranial radiotherapy was performed once at the indicated time point using a Gulmay 200 kV X-ray unit at 1 Gy/minute at room temperature [39]. Neurological symptoms were assessed daily according to the cantonal veterinary office Zurich guidelines. For assessment of early histological changes, animals were euthanized when the first animal showed a grade 2 manifestation of disease progression, including neurological symptoms. Total mRNA or protein lysates were prepared from 20 to 25 mg brain tissue collected at the time of sacrifice from the left and right hemispheres without or with the tumor as indicated. Since SMA-560 tumors were well circumscribed and vascularized, they could be identified, isolated and analyzed separately from the rest of the right hemisphere.

For tumor infiltrating lymphocyte (TIL) isolation, 20,000 GL-261 wildtype or *Met* deficient tumor cells were stereotactically implanted into the right striatum of immunocompetent C57BL/6 mice. Mice were treated with tepotinib at days six to eight and with local radiotherapy at day seven and TIL were isolated from the tumor-bearing hemisphere as described previously [17].

### Flow cytometry

For cell death analysis, cells were stained with Pacific blue-labeled annexin V from Biolegend (San Diego, CA, USA) and propidium iodide (PI) (Sigma-Aldrich) for 15 min in the dark and analyzed by flow cytometry (BD FACSVerse, BD Biosciences). For cell cycle analysis, the cells were fixed with ice-cold 70% ethanol, stained with a solution containing PI, RNase A and Triton X-100 (all Sigma-Aldrich) in PBS for 30 min at 4 °C and then analyzed by flow cytometry (BD FACSVerse analyzer, BD, Allschwil, Switzerland).

For TIL analysis, samples were preincubated with anti-mouse CD16/CD32 (BioLegend) to block Fc receptors and dead cells were excluded by Zombie Aqua staining (Biolegend). The following antibodies were used: anti–CD45-Pblue, anti–CD4-AF700, anti–NKp46-APC, anti–CD11b-PE and anti-F4/80-APC-Cy7 from BioLegend (San Diego, CA) or anti–CD8-PE (BD Pharmingen), and isotype-matched antibodies from Sigma-Aldrich. Data acquisition was done on a BD FACSVerse and data analysis with FlowJo (Tree Star, Stanford, CA, USA).

### Cytotoxicity assay

Syngeneic splenocytes were isolated from C57BL/6 mice and activated with 1 μg/mL Concanavalin A (Sigma-Aldrich) for 20 h and then maintained in RPMI1640 (Gibco Life Technologies) supplemented with 10% FCS, 10 mmol/L HEPES, 2 mmol/L l-glutamine, 1 mmol/L pyruvate, 0.1 mmol/L nonessential amino acids (all from Gibco), 50 μmol/L 2-mercaptoethanol (Sigma-Aldrich), and 25 IU/mL recombinant murine IL2 (PeproTech) for 5–8 days and subsequently used as effector cells. Glioma cells, pretreated with tepotinib or irradiation as indicated for 24 h, were labeled with PKH26 (Sigma-Aldrich) and then used as target cells and cocultured for 20 h with splenocytes at different effector: target ratios. Glioma cell lysis was assessed by an flow cytometry–based assay upon live/dead staining with Zombie-NIR (Biolegend). Specific lysis was expressed as percentage of dead labeled target cells.

### Histology and immunohistochemistry

Sections (8 μm thick) of tumor-bearing cryopreserved brains were prepared. The mean tumor size was determined by measuring the largest tumor area in the horizontal plane on hematoxylin and eosin (H&E)-stained sections from a low-magnification (2.5x) image and the longest perpendicular diameters using the ellipsoid geometric primitive formula [38]. Brain sections were immunostained with Ki-67 antibody (clone SP6, Epitomics, Burlingame, CA), anti-mouse CD31 antibody (clone MEC 13.3, BD Pharmingen, Franklin Lakes, NJ), anti-CD45 antibody (clone 30-F11; Biolegend), anti-CD3 (clone 17A2; BD Biosciences, San Jose, CA) or anti-CD11b (clone M1/70; BD Biosciences) and histofine simple stain mouse MAX PO anti-rabbit secondary antibody (Nichirei Biosciences, Tokyo, Japan) to determine proliferation. Histofine simple stain mouse MAX PO anti-rat (Nichirei Biosciences) and DAB chromogen were used to stain blood vessels. The percentage of Ki-67 − positive tumor nuclei and the mean number of CD31-positive intratumoral vessels per high power field (× 40 objective) were calculated using three randomly selected different microscopic fields of each section for three 3 mice per group or the DAB-positive signals were quantified using the ImageJ software (https://imagej.nih.gov/) [12].

### Statistical analysis

All in vitro and in vivo experiments reported here were performed in biological replicates, i.e., in independent experiments and with different passage numbers of cell lines. Statistical significance was assessed by two-sided unpaired t-test or two-way ANOVA with Bonferroni post-testing (GraphPad Prism). Tumor size was analyzed using the Mann–Whitney U non-parametric test. Survival was analyzed using a Kaplan–Meier survival plot followed by a log-rank (Mantel-Cox) test. Differences were considered statistically significant when the p value was below 0.05.

## Results

### *Characterization of the HGF/MET axis in mouse glioma models *in vitro

**Fig. 1:**
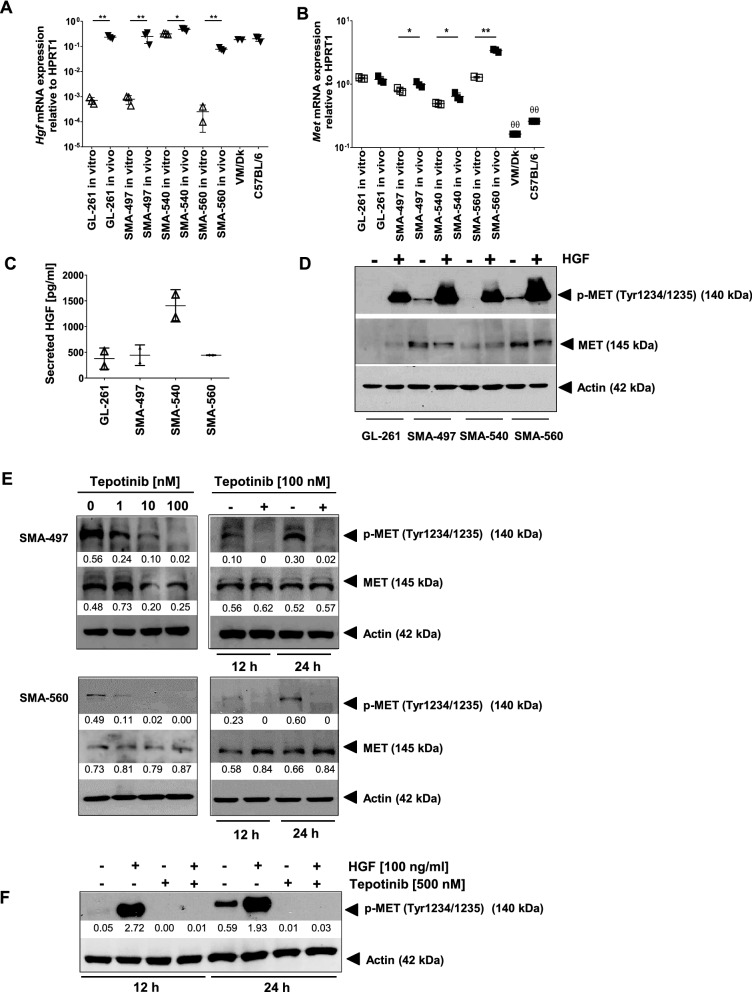
HGF/MET pathway activity in murine glioma cells. A,B. *Hgf* and *Met* mRNA expression were assessed by RT-PCR in cell lines, tumor or healthy brain tissue (^*^ *p* < 0.05, ^**^ *p* < 0.01, ^ѲѲ^ *p* < 0.01 tumor versus healthy brain). C,D. HGF protein release and constitutive or HGF-stimulated p-MET levels were assessed by ELISA (HGF) (C) or immunoblot (p-MET) in cell lines in vitro (D). The intensities of protein bands relative to actin were quantitated using ImageJ Gel Analysis. E. SMA-497 or SMA-560 cells were exposed to tepotinib to determine time- and concentration-dependent inhibition of MET phosphorylation. F. SMA-560 cells were exposed to tepotinib at 100 nM or HGF at 50 ng/ml or both for 12 or 24 h and MET phosphorylation was determined by immunoblot

We first examined HGF and MET expression levels and constitutive MET activation in the GL-261, SMA-497, SMA-540 or SMA-560 mouse glioma models. All four models expressed *Hgf* and *Met* mRNA in vitro and in vivo. We observed significantly higher *Hgf* mRNA levels in tumor and normal brain samples than in the cell lines, indicating that HGF is mainly expressed by stroma cells in both, normal brain and tumors. In contrast, *Met* mRNA expression was significantly upregulated in tumor tissues compared to normal brain tissue (Fig. 1A, B). Flow cytometry-based analysis of the GL-261 tumor-bearing hemisphere demonstrated that MET is expressed by tumor and brain parenchymal cells (Additional file 1: Fig. S1). *Hgf* mRNA expression and HGF protein release in vitro were highly correlated (*r = *0.998, *p = *0.002) among the four mouse cell lines, with SMA-540 demonstrating the highest HGF expression (Fig. 1C). Constitutive MET phosphorylation was detected in SMA-497 and SMA-560 cells and all cell lines accumulated p-MET in response to exogenous HGF (Fig. 1D). Next, we confirmed that exposure of SMA-497 and SMA-560 cells to the MET inhibitor, tepotinib, suppressed MET phosphorylation in a time- and concentration-dependent manner (Fig. 1E). Similarly, tepotinib abrogated the HGF-induced accumulation of p-MET (Fig. 1F). Yet, there were no growth inhibitory effects of tepotinib [33] at concentrations up to 1 µM in acute growth inhibition or clonogenicity assays (Additional file 1: Fig. S1). In summary, the MET pathway is active, but not essential for survival in any of the tested cell lines.

### *Induction of HGF/MET signaling upon irradiation in the SMA-560 model, but no radiosensitization by inhibition of HGF/MET signaling *in vitro

**Fig. 2:**
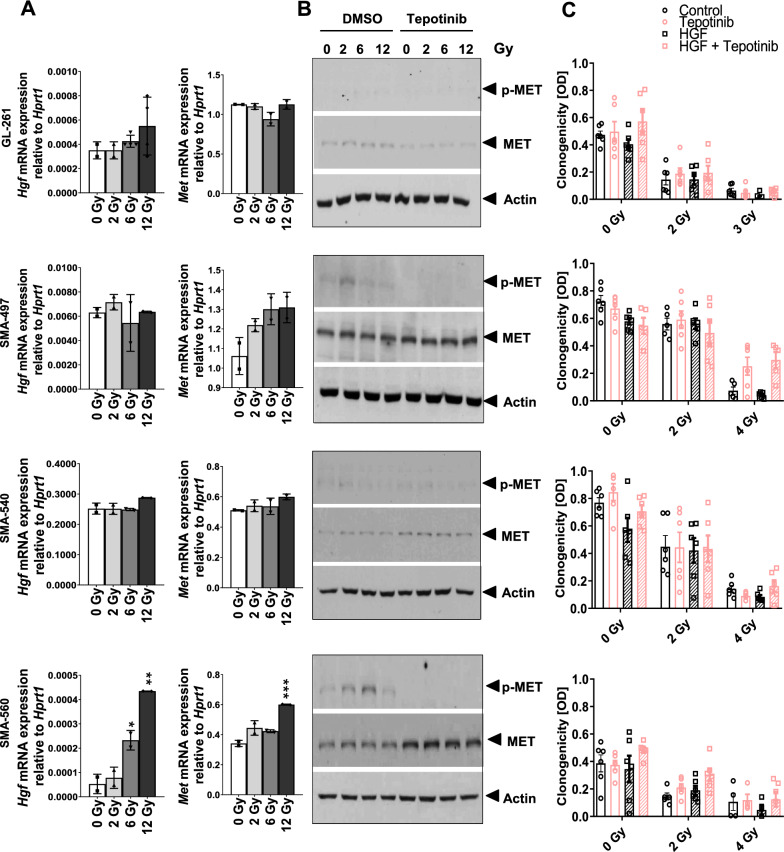
Modulation of the HGF/MET pathway by irradiation in vitro. A,B. The cells were untreated or exposed to irradiation in the absence or presence of tepotinib as pre (8 h)- and co-treatment (100 nM), and 24 h later assessed for expression of Hgf and Met mRNA (A) or for MET and p-MET levels by immunoblot (B). C. Cells were exposed to irradiation in the absence or presence of tepotinib at 100 nM or HGF at 50 ng/ml or both and were then monitored for clonogenic survival (n = 6, mean and SEM; * p < 0.05, **p < 0.01, *** p < 0.001 relative to control)

There were no major changes in *Hgf* or *Met* mRNA expression upon irradiation in GL-261, SMA-497 or SMA-540 cells. In contrast, there was an irradiation dose-dependent induction of *Hgf* and *Met* mRNA expression in SMA-560 cells (Fig. 2A) which translated into tepotinib-sensitive MET phosphorylation (Fig. 2B). Yet, at concentrations known to specifically inhibit MET phosphorylation (Fig. 1E), tepotinib failed to sensitize glioma cells to the inhibitory effects of irradiation in vitro (Fig. 2C). To rule out that this negative effect was merely the result of low availability of HGF in the cell culture setting, similar experiments were conducted in the presence of exogenous HGF, but again no sensitization to irradiation by tepotinib became apparent (Fig. 2C). Thus, independently of MET pathway activation upon irradiation, there was no synergy between irradiation and MET inhibition in vitro*.*

### *Tepotinib inhibits basal and irradiation-induced activation of murine glioma cell invasiveness *in vitro

**Fig. 3:**
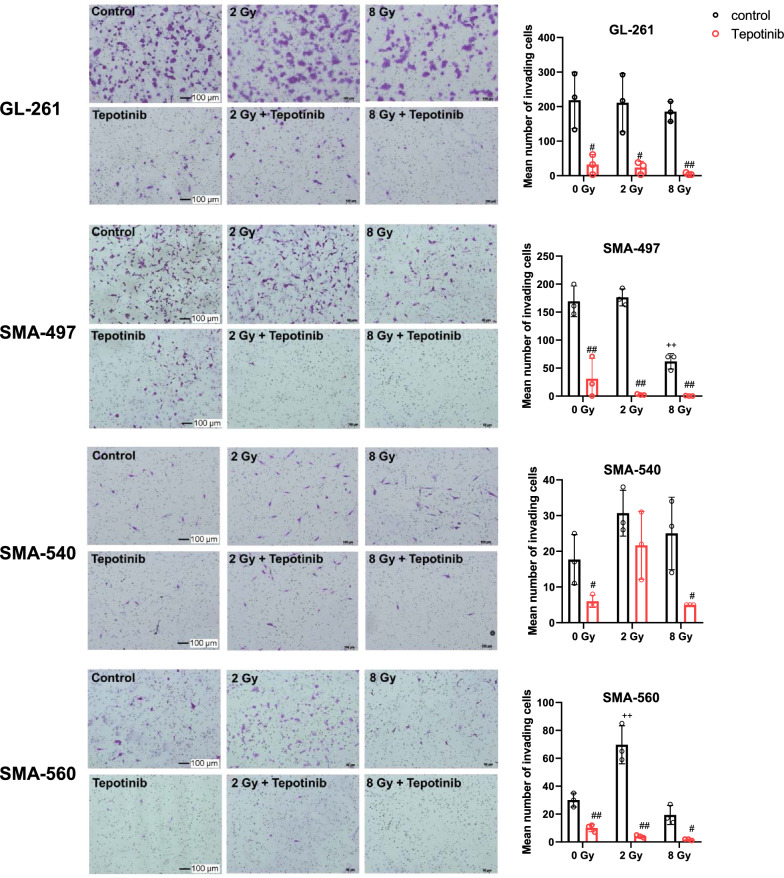
Suppression of invasion of mouse glioma cells by MET inhibition in vitro. Invasiveness was assessed in Corning biocoat matrigel invasion chambers in the absence or presence of tepotinib as pre (8 h)- and co-treatment (100 nM) and without or with irradiation at 2 or 8 Gy. At 24 h after irradiation, equal number of viable cells were re-suspended in fresh serum-free DMEM supplemented or not with tepotinib. The mean number of invading cells was determined 22 h later (^+^*p* < 0.05, effect of irradiation; ^#^*p* < 0.05, effect of tepotinib). The scale bar represents 100 µm

Next, we evaluated the motogenic activity of HGF/MET in response to irradiation in all cell lines in vitro. Low-dose irradiation (2 Gy) increased the invasiveness of SMA-540 and SMA-560 cells whereas high-dose irradiation (8 Gy) reduced invasiveness in SMA-497 and SMA-560 cells. Tepotinib suppressed invasiveness in all cell lines without or with irradiation, except in low dose-irradiated SMA-540 cells (Fig. 3).

### *Synergistic growth inhibition of murine gliomas by radiotherapy and tepotinib *in vivo

**Fig. 4:**
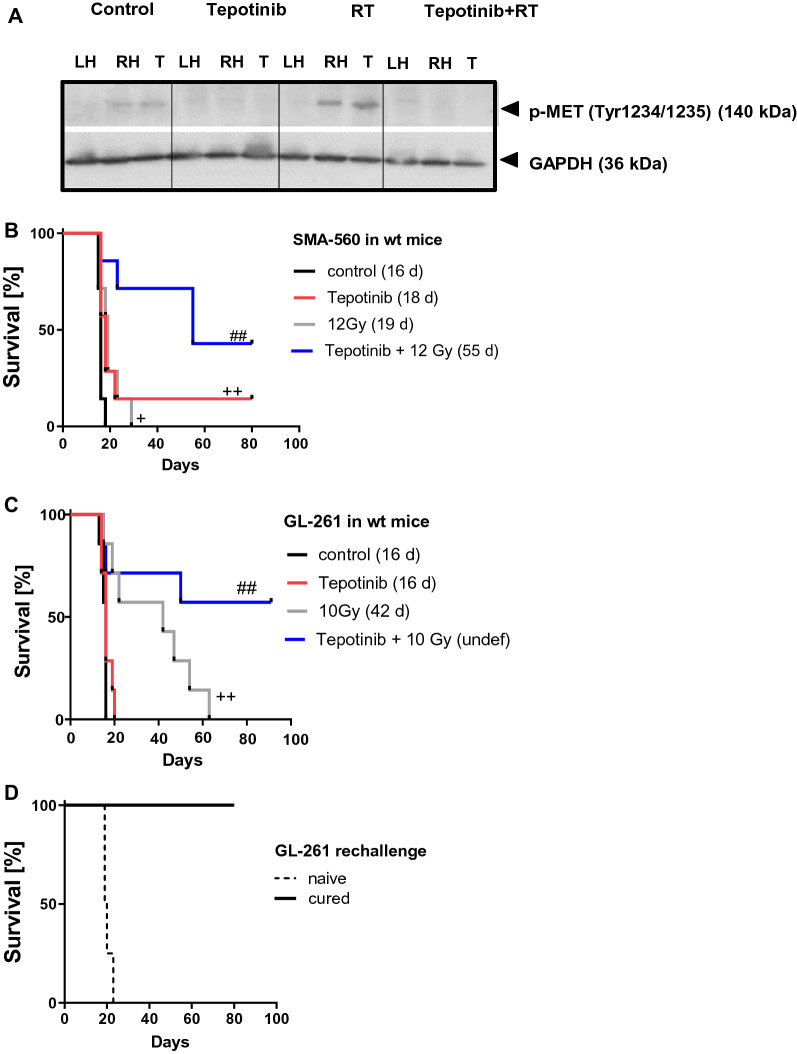
Synergistic prolongation of survival by irradiation and tepotinib-mediated MET inhibition in the SMA-560 glioma model in vivo*.* A,B. Syngeneic mice were intracranially implanted with SMA-560 glioma cells and treated daily with 100 mg/kg tepotinib from day 6 on or solvent, or with a single dose of 12 Gy on day 7, or in combination. A. Tumor lysates pooled from 2 pre-randomized animals per group were analysed by immunoblot for target inhibition (p-MET) (LH, left hemisphere; RH, right hemisphere without tumor; T, tumor removed from the right hemisphere). B. Kaplan–Meier survival curve (^+^ *p* < 0.05, ^++^ *p* < 0.01, versus control; ^##^ *p* < 0.01, versus irradiation). C,D. Syngeneic mice were intracranially implanted with GL-261 glioma cells and treated daily with 100 mg/kg tepotinib from day 6 on or solvent, or with a single dose of 10 Gy on day 7, or the combination of both. C. Kaplan–Meier survival curves were analyzed via log-rank test (^++^ *p* < 0.01, versus control; ^##^ *p* < 0.01, versus irradiation). D. Mice surviving in (C) were re-challenged after 13 weeks after initial tumor cell injection with GL-261 cell implantation into the contralateral hemisphere. Naïve mice implanted with GL-261 cells were used as controls. Kaplan–Meier survival curves are shown

Based on high *Met* gene expression and constitutive MET phosphorylation in the tumor tissue (Fig. 1B, Additional file 1: Fig. S2), the SMA-560 model was selected to assess the effects of radiotherapy in combination with pharmacologic inhibition of MET by tepotinib in vivo. We first confirmed target inhibition by tepotinib by measuring p-MET levels in tumor samples by immunoblot: p-MET was detected in the tumor and the tumor-bearing right hemisphere. It was induced by irradiation, but no p-MET was detected in animals treated with tepotinib (Fig. 4A). Neither tepotinib alone nor radiotherapy alone had a major effect on survival, but their combination resulted in a strong synergistic prolongation of survival from around 20 days without or with monotherapy to around 60 days with the combination. In the control and the radiotherapy groups, all mice were euthanized because of symptoms related to tumor growth. In contrast, one mouse in the tepotinib group and three mice in the combination group were alive and free from tumor at day 80 (Fig. 4B). We then studied a second model to validate and extend these observations. In the GL-261 model, selected for its C57Bl/6 background, radiotherapy, but not tepotinib alone, had a major effect on survival. Again, combination therapy resulted in a prominent survival prolongation compared to either treatment alone. There were approximately 50% long-term survivors in the co-treatment group (Fig. 4C). The long-term surviving mice were rechallenged with GL-261 cells at 90 days and all 4 animals remained asymptomatic until termination of the experiment after another 50 days (Fig. 4D). Thus, in contrast to the in vitro data (Fig. 2C), we observed strong synergy of irradiation and tepotinib in both syngeneic glioma models in vivo.

### Cellular and molecular mechanisms mediating synergistic growth inhibition of experimental gliomas by irradiation and tepotinib

**Fig. 5:**
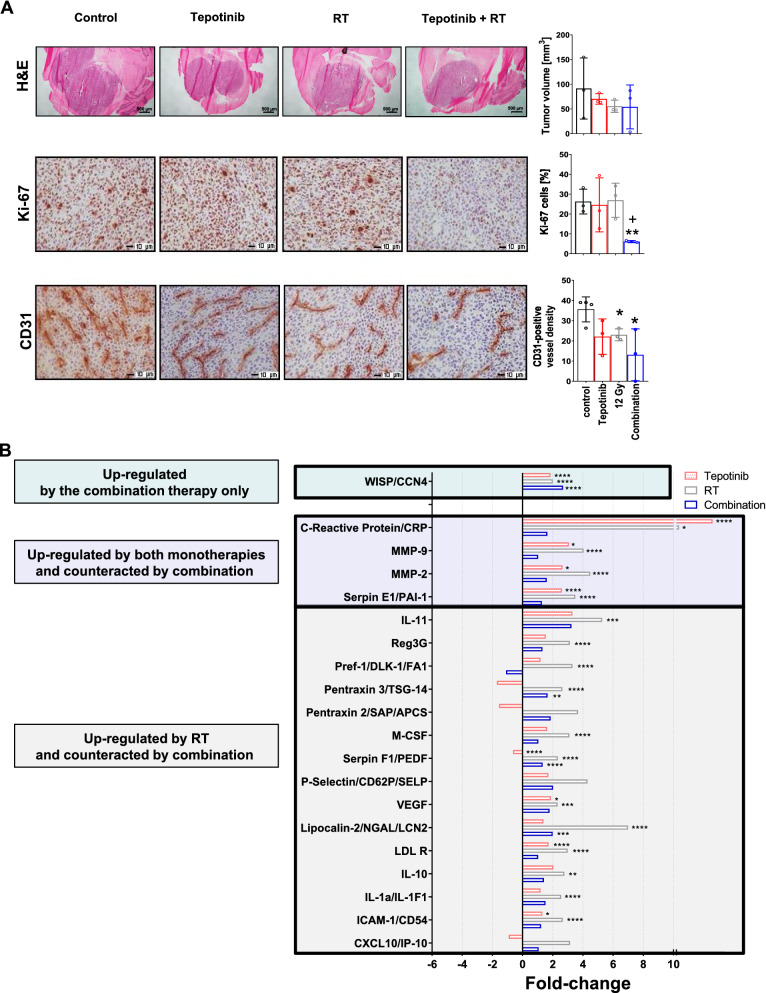
Cellular and molecular responses to tepotinib, irradiation or their combination. A,B. Syngeneic mice were intracranially implanted with SMA-560 glioma cells and treated daily with 100 mg/kg tepotinib from day 6 on or solvent, or with a single dose of 12 Gy on day 7, or in combination. A. Brains from three pre-randomized animals per group were stained for H&E (upper row), Ki-67 (middle row) or CD31 (lower row). Sections were counterstained with hematoxylin (blue). Quantification of immunoreactivity is shown in the right panels (*n* = 3, * *p* < 0.05, t-test, compared with control). B. Tumor lysates pooled from 2 prerandomized animals per group were analysed by proteome profiler array. Fold-changes indicate down-regulation (left) or up-regulation (right) versus control tumors. A difference down or up of twofold versus control was used as cut-off to assign a target to any group. Not all groups shown in Table S1 are included here

To gain insight into the mode of action mediating synergy in vivo, we performed tissue analysis using morphological and immunohistochemical assessments from mice per a randomization list when the first mouse in any group became symptomatic. Animals in all treatment groups showed a reduction in tumor volume by trend relative to untreated controls. Yet, while the percentage of Ki-67-stained nuclei remained unaffected in both monotherapy groups, there was a strong decrease of Ki-67-positive cells in the combination group (Fig. 5A). No such effect of the combination was observed when the same treatments were administered acutely in vitro (Additional file 1: Fig. S3). SMA-560 cells give rise to highly vascularized tumors reflected by high density of CD31-positive vessels [1]. Here, we observed a reduction in CD31 immunoreactivity in both monotherapy arms (1.8-fold) and more so in the combination arm (2.8-fold) (Fig. 5A, bottom).

Since we observed synergy of growth inhibition in vivo, but not in *vitro*, we concluded that the microenvironment might contribute to the therapeutic effect. Accordingly, we assessed differences in host cell infiltration in tumors from the four treatment groups, but observed no consistent differences at early timepoints, that is, when the first mice became symptomatic (data not shown). We then compared the cytokine profiles in tumor tissue lysates ex vivo in the 4 treatment arms. We first noted that almost all cytokines were induced rather than decreased by any therapeutic measure; only tepotinib monotherapy reduced the levels of some cytokines, whereas irradiation alone produced the most prominent induction of cytokines. The only cytokine that was induced by the combination, but not by either monotherapy alone, was the WNT1-inducible signaling pathway protein (WISP/CCN4). The likely most relevant pattern was an induction upon irradiation that was attenuated by tepotinib. This group of molecules included mediators of angiogenesis and pro-inflammatory cell adhesion molecules including VEGF, intercellular adhesion molecule 1 (ICAM-1 / CD54), serpinE1/PAI-1, P-selectin and the matrix metalloproteinases (MMP) -2 and -9, the antiangiogenic and anti-tumorigenic factor SerpinF1/PEDF, mediators of the innate immune response such as lipocalin-2, the colony stimulating factor 1 M-CSF and the innate pattern recognition molecules pentraxin 2/3 and C-reactive protein (CRP, pentraxin 1), pro-inflammatory cytokine IL-1a/IL-1F1 and CXCL10; the anti-inflammatory cytokines IL-10 and IL-11, the member of the notch/delta/serrate protein family pref-1/DLK-1/FA1, and low-density lipoprotein receptor (LDL-R) (Fig. 5B, Additional file 1: Table S1). These data suggest that Met inhibition counteracts the induction of proinflammatory, immunomodulatory and proangiogenic molecules by irradiation and allows radiotherapy to be more effective.

### Synergistic suppression of glioma growth by irradiation and tepotinib requires adaptive immunity and MET expression in the tumor

**Fig. 6:**
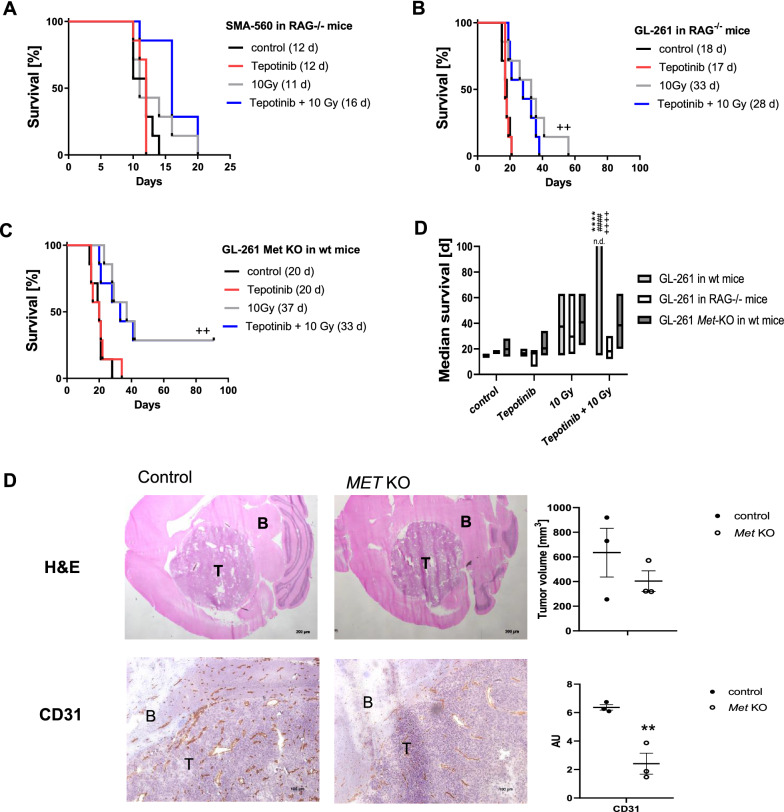
Modulation of response to tepotinib and irradiation alone or in combination by loss of adaptive immunity. A. *Rag1*^*−/−*^ mice were intracranially implanted with SMA-560 glioma cells, treated daily with 100 mg/kg tepotinib from day 6 on or solvent, or with a single dose of 10 Gy on day 7, or in combination. Kaplan–Meier survival curves are shown. B,C. GL-261 wildtype cells were intracranially implanted into C57/BL6 *Rag1*^*−/−*^ mice (B), or GL-261 *Met* knockout cells were intracranially implanted into C57/BL6 wildtype mice (C). Mice were treated daily with 100 mg/kg tepotinib or solvent from day 6 on, or with a single dose of 10 Gy on day 7, or a combination thereof, and Kaplan–Meier survival curves are shown (^++^ *p* < 0.01, versus control). D. Illustration of median survival differences between treatment groups in the GL-261 model (n.d., not defined, > 50% of animals cured; ^***^ *p* < 0.0001, versus control; ^###^ *p* < 0.0001, versus tepotinib; ^+++^ *p* < 0.0001, versus radiotherapy). Symptomatic animals implanted with GL-261 wildtype or *Met* knockout cells were euthanized, the brains removed, cut into thin slices and stained with H&E or for CD31 (T, tumor; B, brain). Representative images are shown (left), stainings were quantified using Image J (right) (^**^ *p* < 0.01, relative to control; AU, arbitrary units)

To confirm a role of the tumor microenvironmental in mediating the synergistic response of experimental gliomas to the combination of radiotherapy and tepotinib, we explored the combination therapy in immunodeficient animals. Here we noted that the synergistic effect of combination therapy was strongly attenuated when SMA-560 cells were grown in *Rag1*^−/−^ mice that lack mature B and T cells (Fig. 6A). Likewise, when GL-261 cells were grown in *Rag1*^−/−^ mice, irradiation alone retained its activity, but synergy with tepotinib was lost in this model, too (Fig. 6B).

Finally, to dissect tumor microenvironmental *versus* tumor-autonomous mechanisms in mediating the synergistic response of experimental gliomas to the combination of radiotherapy and tepotinib, we disrupted the *Met* gene in GL-261 glioma cells by CRISPR/Cas9 technology. As a consequence, *met* mRNA levels were not further detected in the MET-deficient GL-261 cells in vitro and, in contrast to wildtype cells, MET-deficient GL-261 did not respond with MET phosphorylation to exogenous HGF exposure. Similarly, accumulation of p-AKT upon stimulation with HGF was observed only in wildtype, but not in MET-deficient cell lines (Additional file 1: Fig. S4). Moreover*,* MET-deficient GL-261 glioma cells had no growth disadvantage as determined by measures of cell doubling time, viability or cell cycle progression in vitro (Additional file 1: Fig. S4). In vivo, irradiation was still active when *Met* was disrupted in the tumor and even cured 2 out of 7 mice, but synergy with tepotinib was also lost in this model (Fig. 6C). Comparative survival data are compiled in Fig. 6D. Immunohistochemical assessments of the mouse brains revealed reduced tumor volumes by trend, and a significant reduction in CD31-positive vessels in MET-deficient tumors relative to controls (Fig. 6E). In contrast, the proportion of Ki67-positive cells and the number of tumor-infiltrating lymphocytes remained unaffected at a late stage, when each animal was symptomatic (Additional file 1: Fig. S5). Thus, synergy of irradiation and MET inhibition appears to require an intact immune system and MET expression in the tumor.

### MET inhibition counteracts irradiation-induced activation of TGF-β signaling in experimental gliomas

**Fig. 7:**
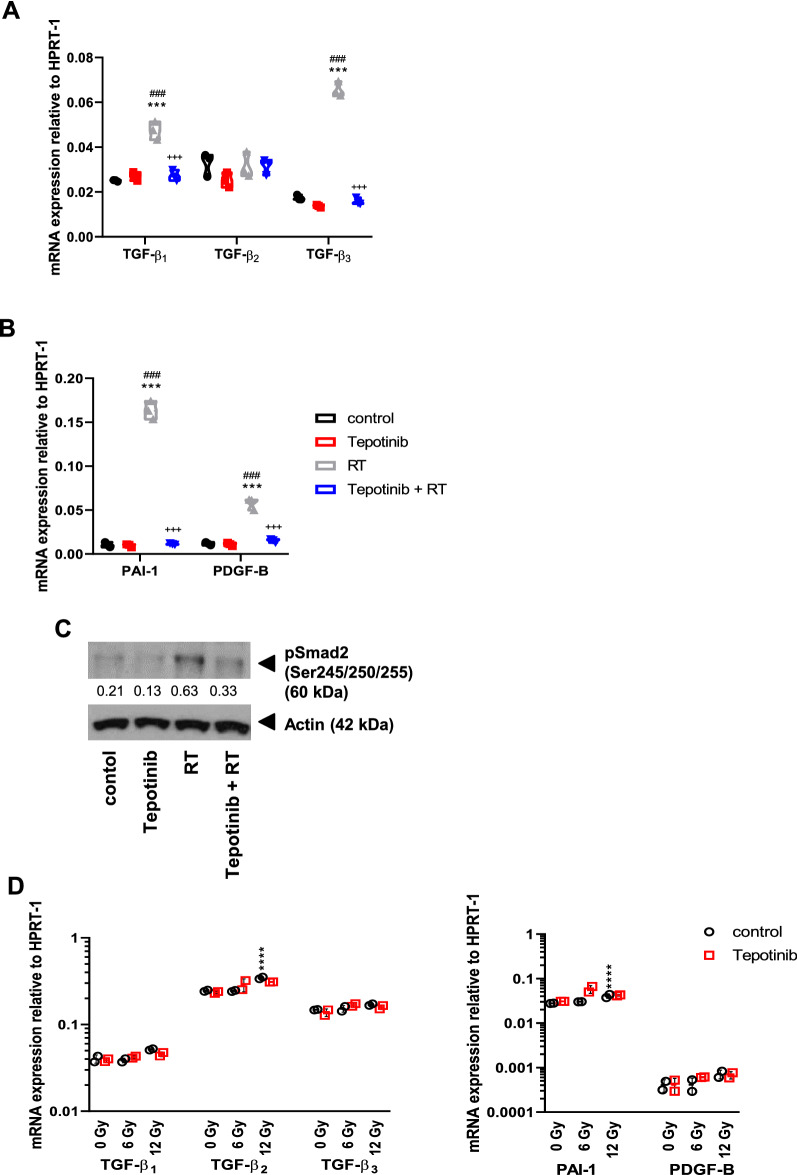
Modulation of TGF-β pathway activity in response to tepotinib and irradiation alone or in combination. Syngeneic mice were intracranially implanted with GL-261 glioma cells and treated daily with 100 mg/kg tepotinib from day 6 on or solvent, or with a single dose of 10 Gy on day 7, or in combination. RNA and protein were extracted from the right tumor-bearing hemispheres and analyzed for TGF-β pathway activity. A,B. *TGF-β1*, *TGF-β2*, *TGF-β3*, *PAI-1* or *PDGF-B* mRNA expression levels relative to HPRT1 levels were assessed by RT-PCR (^***^ *p* < 0.0001, versus control; ^###^ *p* < 0.0001, versus tepotinib; ^+++^ *p* < 0.0001, versus radiotherapy). C. pSMAD2 protein levels were assessed by immunoblot. The intensities of protein bands relative to actin were quantified using ImageJ Gel Analysis. D. The cells were untreated or exposed to irradiation in the absence or presence of tepotinib as pre (8 h)- and co-treatment (100 nM), and 24 h later assessed for expression of TGF-β_1_, TGF-β_2_, TGF-β_3_, PAI-1 and PDGF-B mRNA levels. Data are presented as mean ± SD (***, P < 0.001, compared to un-irradiated control)

Since the cytokine profiling summarized above had revealed profound changes in a number of cytokines that are positively regulated by TGF-β like VEGF, MMP or PAI-1 [16, 40, 52] (Fig. 5B), we next evaluated a possible role of TGF-β signaling in mediating the synergistic response of experimental gliomas to radiotherapy and tepotinib treatment in vivo. Consistent with studies that report irradiation-induced TGF-β signaling [13, 45], we observed increased mRNA expression of TGF-β_1_ and –β_3_ in GL-261 tumors upon irradiation alone, which was counteracted by concomitant MET inhibition. TGF-β_2_ expresssion remained unaffected by either treatment (Fig. 7A). Similarly, expression levels of the two TGF-β bona fide response genes, PDGF-B and PAI-1, were induced by irradiation alone, but remained unaltered with combinatorial treatment (Fig. 7B). To further confirm the hypothesis that MET inhibition counteracts irradiation-induced activation of TGF-β, we assessed pSMAD2 levels as a surrogate marker of TGF-β pathway activity in tumor lysates. In line with the mRNA data, SMAD2 phosphorlation was induced by radiotherapy, but decreased when tepotinib was combined with radiotherapy (Fig. 7C). In the GL-261 model in vitro, TGF-β_2_ was the predominant isoform, and it was – like its downstream target PAI-1—induced upon irradiation with high-dose irradiation, however, this was not blocked by concurrent MET inhibition (Fig. 7D). In the SMA-560 model, TGF-β_1_ was the major TGF-β isoform, and was—like TGF-β_3_—induced upon high-dose irradiation and to some extent decreased when tepotinib was combined with radiotherapy. Similarly, PAI-1 was induced upon irradiation, but this induction was not rescued by co-exposure to tepotinib (Additional file 1: Fig. S6). Finally, immunoblot analysis revealed an irradiation dose-dependent increase of pSMAD2 levels which was not blocked by tepotinib (Additional file 1: Fig. S6). Interestingly, we also noticed a decrease in basal TGF-β mRNA and pSMAD2 protein levels in MET-deficient GL-261 cells in vitro, further supporting a link between these two oncogenic pathways (Additional file 1: Fig. S6). Accordingly, suppression of the TGF-β pathway activity might significantly contribute to the synergistic growth inhibition of radiotherapy and tepotinib in vivo, in the presence of the tumor microenvironment.

### MET expression in the tumor affects the abundance of tumor-infiltrating lymphocytes

**Fig. 8:**
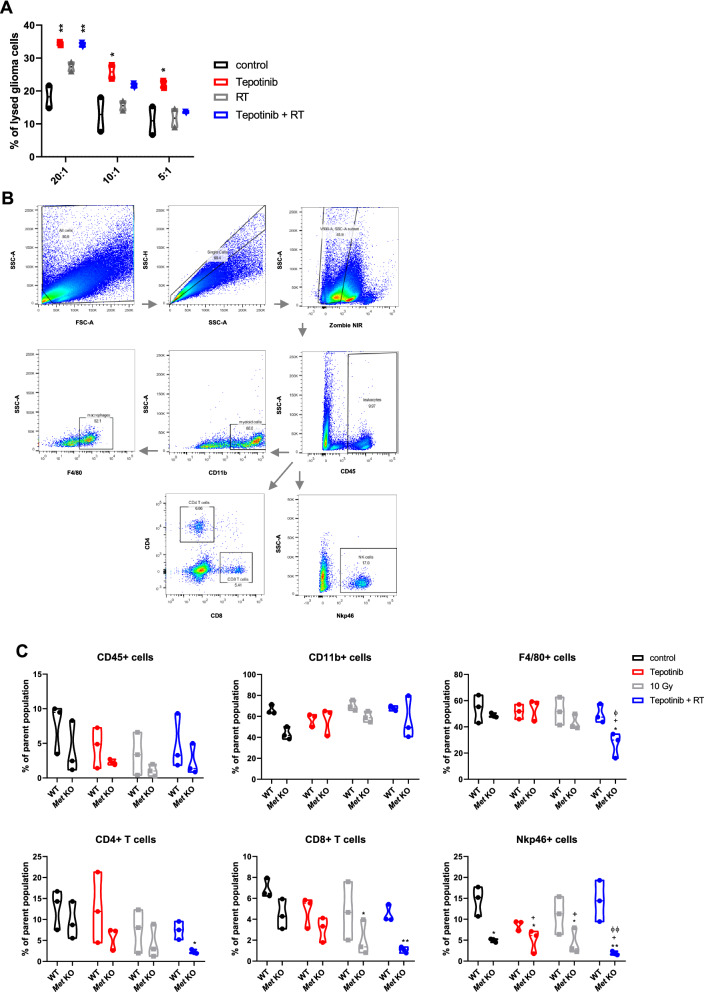
MET expression in the tumor affects the abundance of tumor-infiltrating lymphocytes. A. GL-261 cells, pretreated for 24 h as indicated, were used as target cells in a 20-h cytotoxicity assay. Syngeneic splenocytes were used as effector cells at various effector to target (E:T) ratios. Data are presented as mean ± SD (**, P < 0.01). B-C. GL-261 wildtype or *Met* knockout cells were intracranially implanted into C57/BL6 wildtype mice. Mice were treated with 100 mg/kg tepotinib or solvent from day 6 to 8, or with a single dose of 10 Gy on day 7, or a combination thereof, and tumor-infiltrating lymphocytes were isolated on day 9 from the tumor-bearing hemisphere and analysed by flow cytometry. The gating strategy is shown in B and abundance of each immune cell population in the wildtype versus *Met* KO-deficient tumor-bearing hemisphere for each treatment group is shown in C (^*^ *p* < 0.05, ^**^ *p* < 0.01, versus WT control; ^#^ *p* < 0.05, versus WT tepotinib; ^+^ *p* < 0.05, versus WT 10 Gy; ^φ^* p* < 0.05, ^φ φ^* p* < 0.01, versus WT 10 GyTepotinib + RT)

**Fig. 9:**
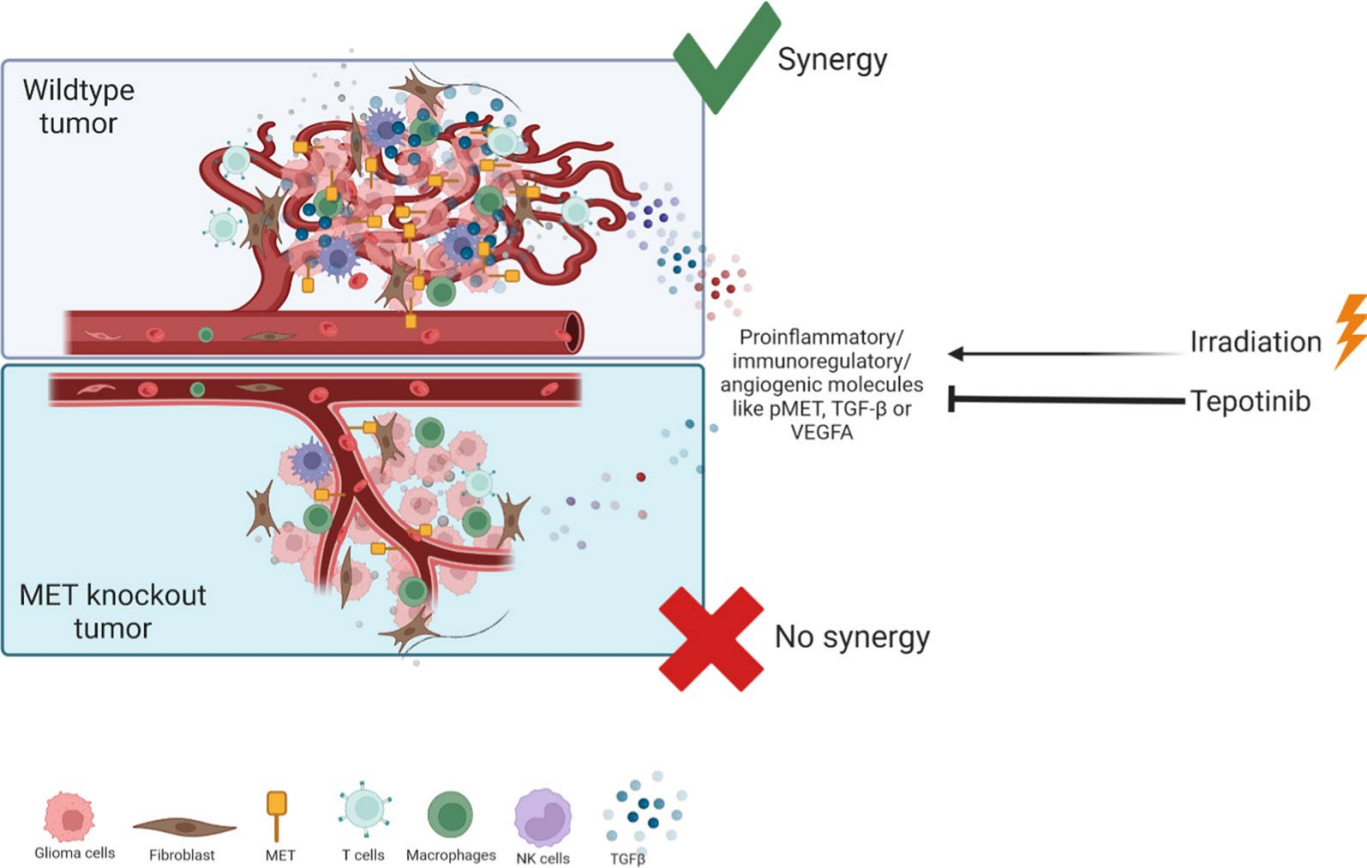
Graphical abstract. *Met* proficient tumors – as opposed to Met deficient tumors—show higher vessel density, increased immune cell infiltration and enhanced expression of proinflammatory, immunomodulatory and proangiogenic molecules in response to irradiation which is counteracted by Met inhibition

To further characterize the mode of action, we performed cytotoxicity assays using splenocytes as immune effectors and GL-261 tumor cells as targets. Here, exposure to tepotinib resulted in significantly enhanced immune cell–mediated cytolysis (Fig. 8A). Finally, we implanted wildtyper or MET-deficient GL-261 tumors in immunocompetent mice and analyzed the immune cells in the tumor microenvironment when treated with or without tepotinib or irradiation or both at an early timepoint by flow cytometry using the gating strategy shown in Fig. 8B. At an early stage, no major differences in immune cell populations were found in response to either treatment in GL-261 wildtype tumors. However, the abundance of lymphoid cells, specifically CD4 and CD8 T cells and natural killer (NK) cells was strongly decreased in *Met* KO tumors, whereas the numbers of myeloid cells were comparable in wildtype and MET-deficient tumors (Fig. 8C). These data suggest that MET-expressing tumors attract more lymphoid cells which might be facilitated by a higher vessel densitiy in wildtype tumors compared with MET-deficient tumors (Fig. 6E). However, the activity of immune cells in wildtype tumors might be attenuated by high TGF-β levels in the tumor microenvironment, especially in response to irradiation. Thus, Met inhibition in established tumors may synergistically contribute to the efficacy of radiotherapy by counteracting its undesirable effects (Fig. 9).

## Discussion

Glioblastoma remains to be one of the most lethal solid cancers. Since the introduction of temozolomide more than 15 years ago [43], no other pharmacological treatment has been shown to prolong survival when added to the standard of care of surgery followed by radiotherapy [47]. Beyond classical cancer chemotherapy, microenvironmental targets such as angiogenesis, infiltration, invasion and immune response have received a lot of interest in the last decade. The HGF/MET pathway is potentially involved in all these processes, and there is strong support from preclinical studies for targeting this pathway in glioblastoma [24, 26].

Here we explored the molecular and cellular mechanisms that mediate synergistic effects of MET pathway inhibition and irradiation in syngeneic mouse models. Although synergistic effects of MET inhibition and irradiation have previously been proposed to be mediated by interference with the cytoprotective role of the MET pathway in the context of DNA repair in human glioma, gastric adenocarcinoma and lung carcinoma cells [15, 31, 48], such mechanisms were unlikely to be operating in our models since there was no synergy between irradiation and pharmacological MET inhibition in vitro (Fig. 2). Conversely, we observed strong synergy of irradiation and tepotinib in two syngeneic glioma models in vivo (Fig. 4). Compared with either treatment alone, combination treatment induced an early suppression of proliferation and of angiogenesis in the SMA-560 model (Fig. 5A). Inhibition of angiogenesis may be a consequence of suppression of irradiation-induced increases in angiogenic molecules such as VEGF, MCSF, MMP2 or MMP9 upon co-treatment with tepotinib (Fig. 5B). It is tempting to speculate that TGF-β is the proximate mediator of these irradiation-induced changes since TGF-β pathway activity was induced by radiotherapy alone, but decreased when tepotinib was combined with radiotherapy (Fig. 7).

Radiation therapy damages cancer cells either directly by DNA damage, followed by cell death, irreversible growth arrest or indirectly by producing free radicals and cytokine-mediated cellular toxicity [35, 46]. Glioblastoma develops radiation resistance by multiple adaptive molecular strategies [3, 15, 41]. Altogether, we report the attenuation by tepotinib of expression of several irradiation-induced cancer-related proinflammatory and immunoregulatory cytokines as a potential mechanism by which tumor growth is delayed by tepotinib when combined with radiotherapy. Complementary studies exploring the efficacy of combination therapy either in immunodeficient mice or in mice carrying tumors with disrupted *Met* gene expression confirmed that synergy requires at least two components, first, expression of MET in the tumor, second, a functional immune system (Fig. 6). If suppression of TGF-β pathway activity was the major therapeutic output of the combined treatment with RT and tepotinib, one may raise the question of why TGF-β inhibition has not been more successful in the clinic. Yet, the majority of clinical trials have explored TGF-β in the recurrent setting without combination with RT [6, 7]. In contrast, preclinical studies suggest synergistic activity of RT and TGF-β inhibition in murine glioma models [21, 22]. There is only limited data on this combination in human glioma patients which were disappointing [51]. However, small molecule TGF-β receptor inhibitors do not exhibit a favorable safety and tolerability and it remains uncertain to what extent TGF-β pathway activity was reduced in these clinical settings at dosing schedules tolerated by human patients [2, 10, 32]. Potentially inhibiting TGF-β activity by removing upstream positive regulators such as MET that are overactive in gliomas represents a preferable approach to interfere with glioma growth than the approaches explored so far.

Limitations of our study include the questionable relevance of our murine models to the human system and a limited dissection of the precise molecular mediators down-stream of MET and TGF-β signaling that are responsible for the synergistic effect of RT and tepotinib. Yet, these preclinical data suggest that MET pathway inhibition in human glioblastoma would be best explored in combination with radiotherapy. Accordingly, the negative outcomes of testing efficacy of the HGF antibody AMG-102 [49] or the MET antibody onartuzumab [11] should not result in abandoning this pathway as a target. The multikinase inhibitor XL-184 (cabozantinib), targeting MET, VEGF-receptor 2 (VEGFR2) and RET, has been evaluated for safety when combined with standard temozolomide chemoradiotherapy in a small cohort of patients with glioblastoma [37], but efficacy data on the combination of RT and MET inhibition in glioblastoma await to be generated.

## Supplementary Information

**Additional file 1**: **Supplementary Table 1**. Patterns of changes in cytokine levels in response to tepotinib, irradiation or combination therapy. A cut-off of 2-fold difference compared to control expression levels was used to assign a target to any group. **Supplementary Figure 1**. MET is expressed by tumor and stromal cells. A. iRFP720-expressing GL-261 were implanted into C57/BL6 mice. The symptomatic animal was euthanized, the brain removed and the single-cell suspension stained for intra- and extracellular MET expression by flow cytometry (blue, isotype control; red, FITC-labeled Met Monoclonal Antibody (eBioclone 7)). B. Murine glioma cells were exposed to tepotinib in acute growth inhibition (left) or clonogenic survival (right) assays. Viability was assessed by MTT assay (* *p*<0.05, ** *p*<0.01, versus control). **Supplementary Figure 2**. MET phosphorylation in healthy mouse brain and tumor-bearing brain. p-MET levels were assessed by immunoblot in protein lysates of brain tissue of healthy or GL-261 glioma-bearing C57Bl/6 mice, or healthy or SMA glioma-bearing VM/Dk mice. Tissue samples were collected at the time of sacrifice of the first symptomatic animals from the left and right hemispheres and from the tumor. **Supplementary Figure 3**. Effects of tepotinib and irradiation on Ki67 expression in mouse glioma cells in vitro. SMA-497 or SMA-560 cells were irradiated at 2 or 12 Gy in the absence or presence of tepotinib at 100 nM (24 h pretreatment) and stained for Ki-67 at 120 h. Data are expressed as percentages of Ki67-positive cells per field of view. **Supplementary Figure 4**. In vitro characterization of MET-deficient GL-261 sublines. MET-deficient sublines of GL-261 were generated by CRISPR/Cas9-based technology. A. Met mRNA levels were assessed by RT-PCR. (** *p*<0.01, versus control). B. P-MET and p-AKT protein levels were assessed by immunoblot. C. Cell doubling times determined by trypan blue staining (left). The number of viable (white) and dead (black) cells was counted daily from day 1 to day 7 (right). D. Viability determined by annexin V/PI staining. E. Cell cycle analysed by flow cytometry 48 h after seeding. **Supplementary Figure 5**. In vivo characterization of MET-deficient GL-261 tumors. MET-deficient sublines of GL-261 were implanted into C57/BL6 mice. Symptomatic animals were euthanized, the brains removed, cut into thin slices and stained for Ki67, CD45, CD3 or CD11b. Representative images are shown (left), stainings were quantified using Image J (right) (** *p*<0.01, relative to control; AU, arbitrary units). **Supplementary Figure 6**. Modulation of the TGF-β pathway by irradiation and Met inhibition in vitro. A-C. SMA-560 cells were untreated or exposed to irradiation as indicated, in the absence or presence of tepotinib as pre (8 h)- and co-treatment (100 nM), and 24 h later assessed for expression of* TGF-β*_1_,* TGF-β*_2_,* TGF-β*_3_, PAI-1 and PDGF-B mRNA (*** *p*<0.001, **** *p*<0.0001 versus control; + *p*<0.05, ++++ *p*<0.0001 versus irradiation alone) (A) or for p-SMAD2 levels by immunoblot (B). pSmad2 levels relative to actin were quantified with ImageJ and are shown in C. D-E. MET-deficient GL-261 sublines, generated by CRISPR/Cas9-based technology, were analysed for * TGF-β*_1_,* TGF-β*_2_, and * TGF-β*_3_ mRNA levels by RT-PCR (** *p*<0.01, relative to control) (D) or for pSMAD2 levels by immunoblot (E).

## Data Availability

All data generated or analysed during this study are included in this published article and its supplementary information files.
