# Supplementary material for: Immunological and tumor-intrinsic mechanisms mediate the synergistic growth suppression of experimental glioblastoma by radiotherapy and MET inhibition
Source: Acta Neuropathol Commun. 2023 Mar 13;11:41. doi: 10.1186/s40478-023-01527-8 (PMC10009975; doi:10.1186/s40478-023-01527-8)
Supplement: Supplementary file 1 — Additional file 1: Supplementary Table 1. Patterns of changes in cytokine levels in response to tepotinib, irradiation or combination therapy. A cut-off of 2-fold difference compared to control expression levels was used to assign a target to any group. Supplementary Figure 1. MET is expressed by tumor and stromal cells. A. iRFP720-expressing GL-261 were implanted into C57/BL6 mice. The symptomatic animal was euthanized, the brain removed and the single-cell suspension stained for intra- and extracellular MET expression by flow cytometry (blue, isotype control; red, FITC-labeled Met Monoclonal Antibody (eBioclone 7)). B. Murine glioma cells were exposed to tepotinib in acute growth inhibition (left) or clonogenic survival (right) assays. Viability was assessed by MTT assay (* p<0.05, ** p<0.01, versus control). Supplementary Figure 2. MET phosphorylation in healthy mouse brain and tumor-bearing brain. p-MET levels were assessed by immunoblot in protein lysates of brain tissue of healthy or GL-261 glioma-bearing C57Bl/6 mice, or healthy or SMA glioma-bearing VM/Dk mice. Tissue samples were collected at the time of sacrifice of the first symptomatic animals from the left and right hemispheres and from the tumor. Supplementary Figure 3. Effects of tepotinib and irradiation on Ki67 expression in mouse glioma cells in vitro. SMA-497 or SMA-560 cells were irradiated at 2 or 12 Gy in the absence or presence of tepotinib at 100 nM (24 h pretreatment) and stained for Ki-67 at 120 h. Data are expressed as percentages of Ki67-positive cells per field of view. Supplementary Figure 4. In vitro characterization of MET-deficient GL-261 sublines. MET-deficient sublines of GL-261 were generated by CRISPR/Cas9-based technology. A. Met mRNA levels were assessed by RT-PCR. (** p<0.01, versus control). B. P-MET and p-AKT protein levels were assessed by immunoblot. C. Cell doubling times determined by trypan blue staining (left). The number of viable (white) and dead (black) cells was co [file 40478_2023_1527_MOESM1_ESM.pptx]

## Slide 1
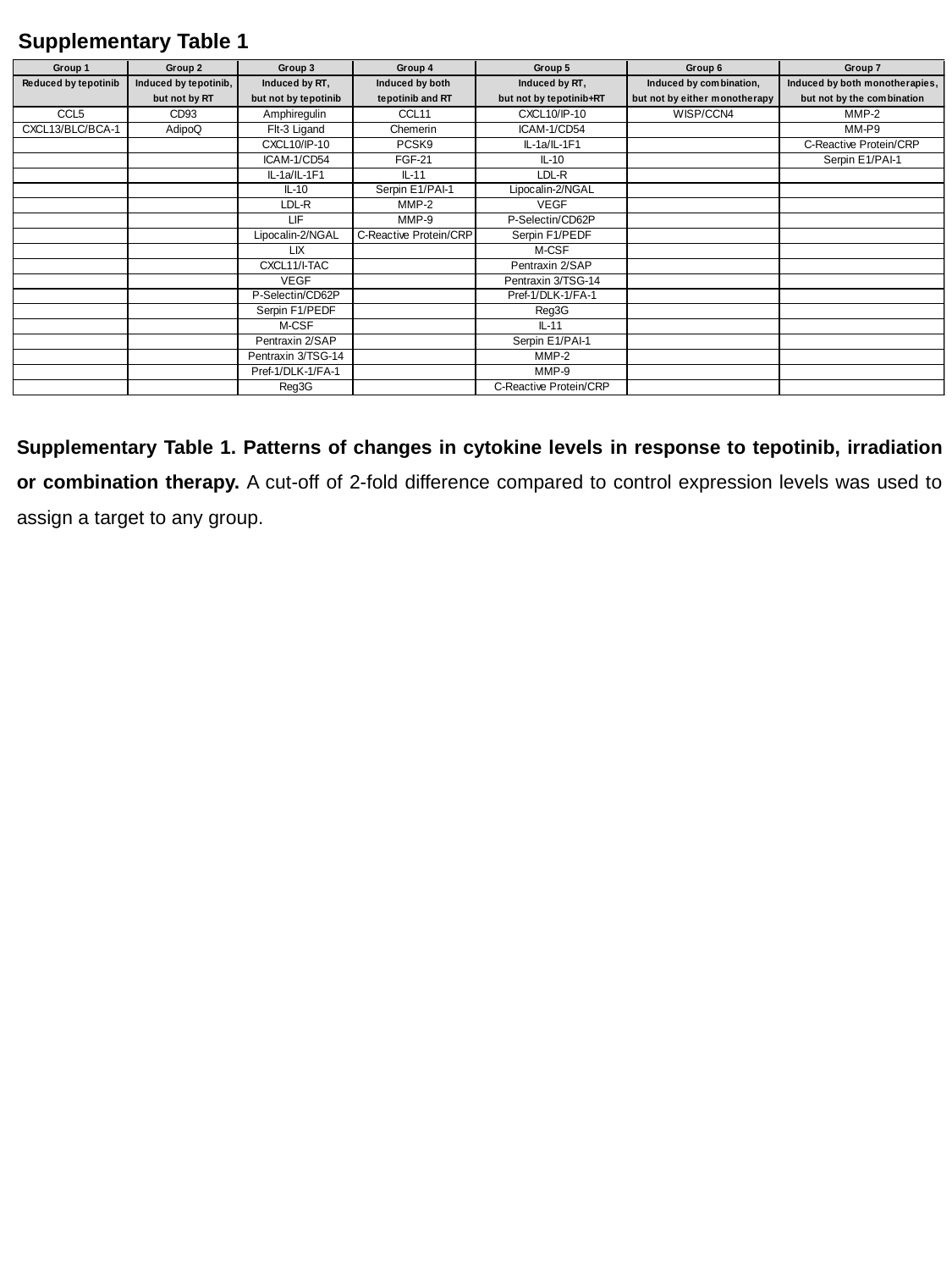

Supplementary Table 1
Supplementary Table 1. Patterns of changes in cytokine levels in response to tepotinib, irradiation or combination therapy. A cut-off of 2-fold difference compared to control expression levels was used to assign a target to any group.

## Slide 2
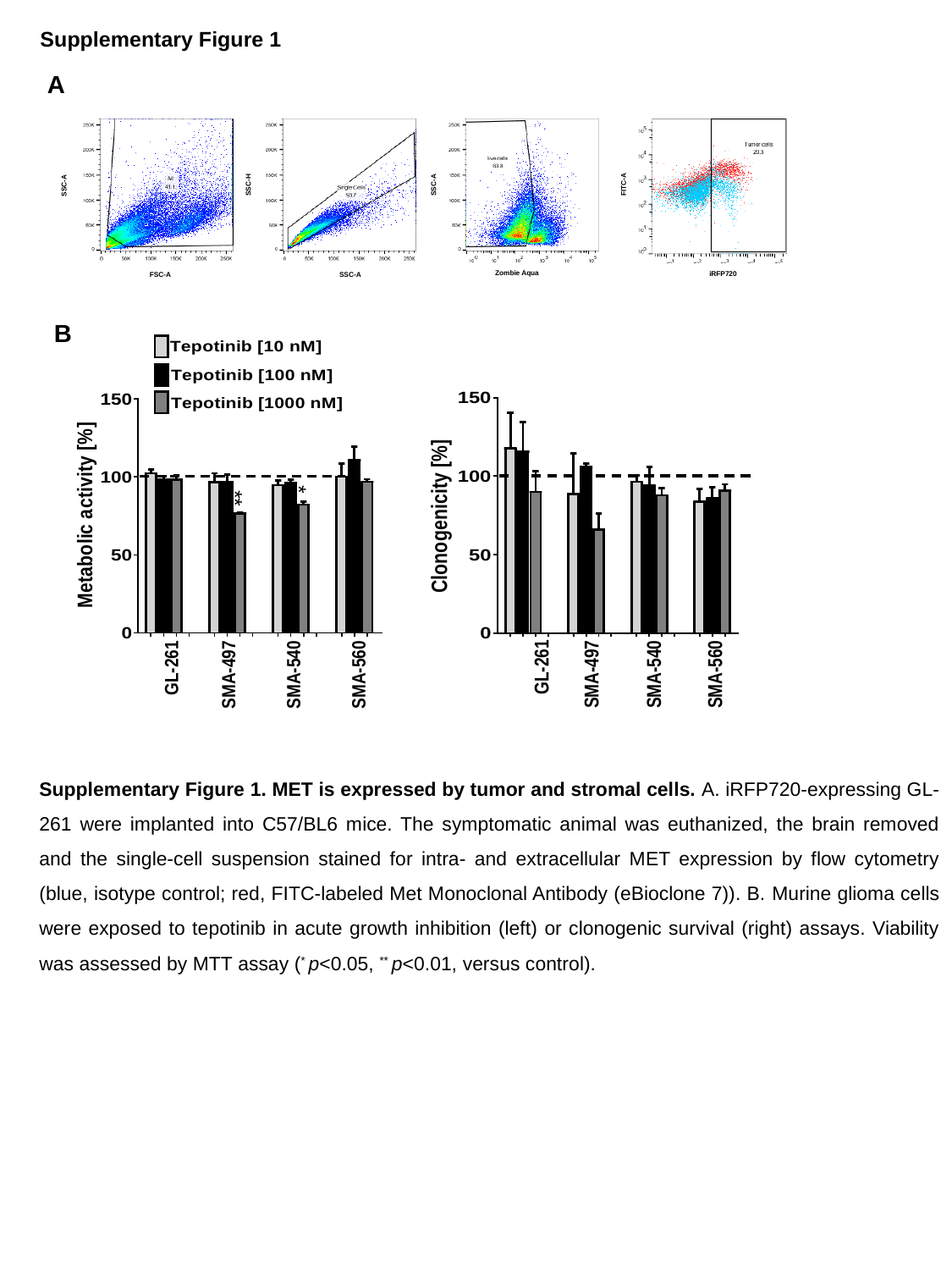

Supplementary Figure 1
A
FITC-A
SSC-H
SSC-A
SSC-A
Zombie Aqua
iRFP720
FSC-A
SSC-A
B
*
**
Supplementary Figure 1. MET is expressed by tumor and stromal cells. A. iRFP720-expressing GL-261 were implanted into C57/BL6 mice. The symptomatic animal was euthanized, the brain removed and the single-cell suspension stained for intra- and extracellular MET expression by flow cytometry (blue, isotype control; red, FITC-labeled Met Monoclonal Antibody (eBioclone 7)). B. Murine glioma cells were exposed to tepotinib in acute growth inhibition (left) or clonogenic survival (right) assays. Viability was assessed by MTT assay (* p<0.05, ** p<0.01, versus control).

## Slide 3
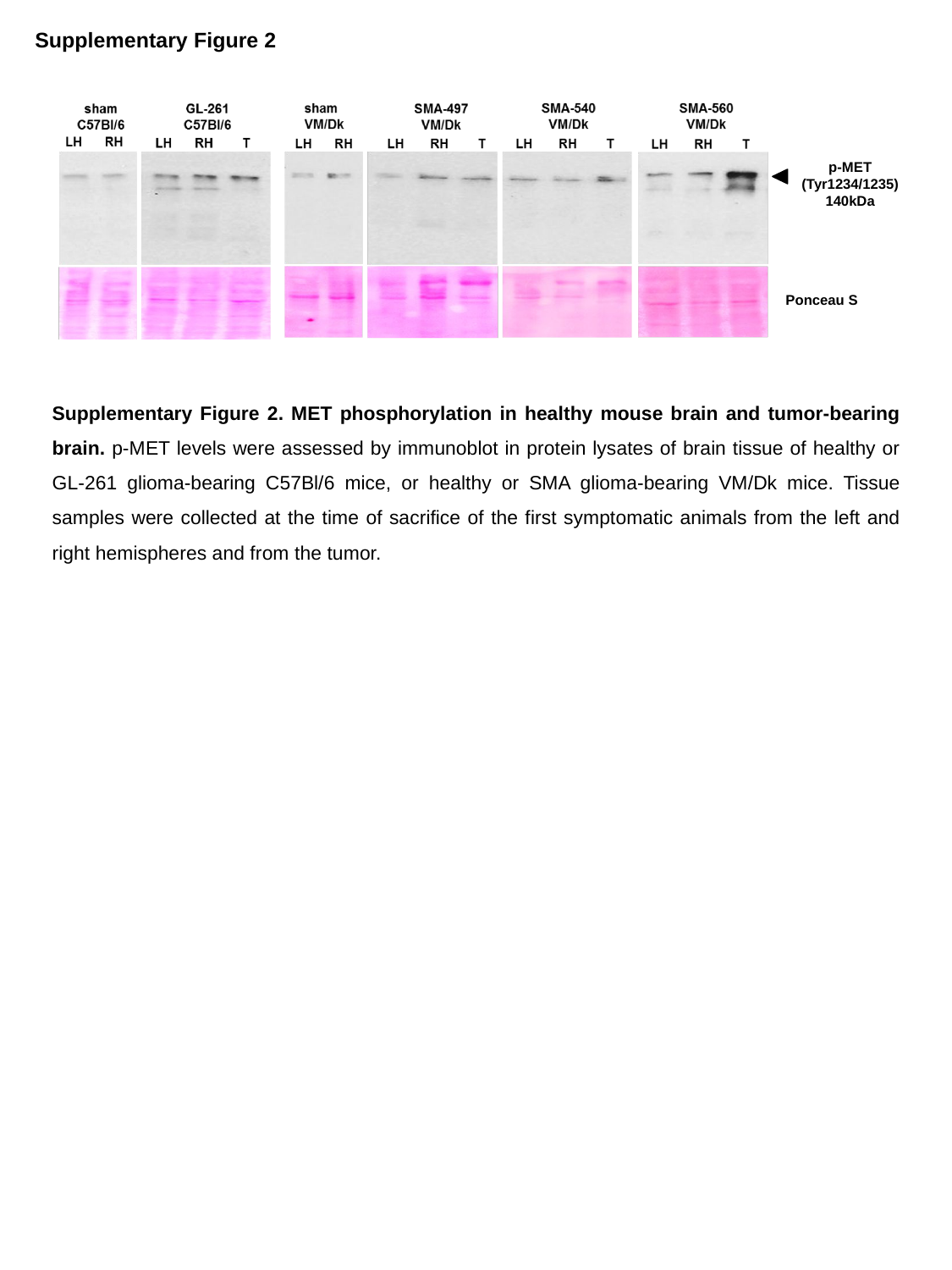

Supplementary Figure 2
p-MET
(Tyr1234/1235)
140kDa
Ponceau S
Supplementary Figure 2. MET phosphorylation in healthy mouse brain and tumor-bearing brain. p-MET levels were assessed by immunoblot in protein lysates of brain tissue of healthy or GL-261 glioma-bearing C57Bl/6 mice, or healthy or SMA glioma-bearing VM/Dk mice. Tissue samples were collected at the time of sacrifice of the first symptomatic animals from the left and right hemispheres and from the tumor.

## Slide 4
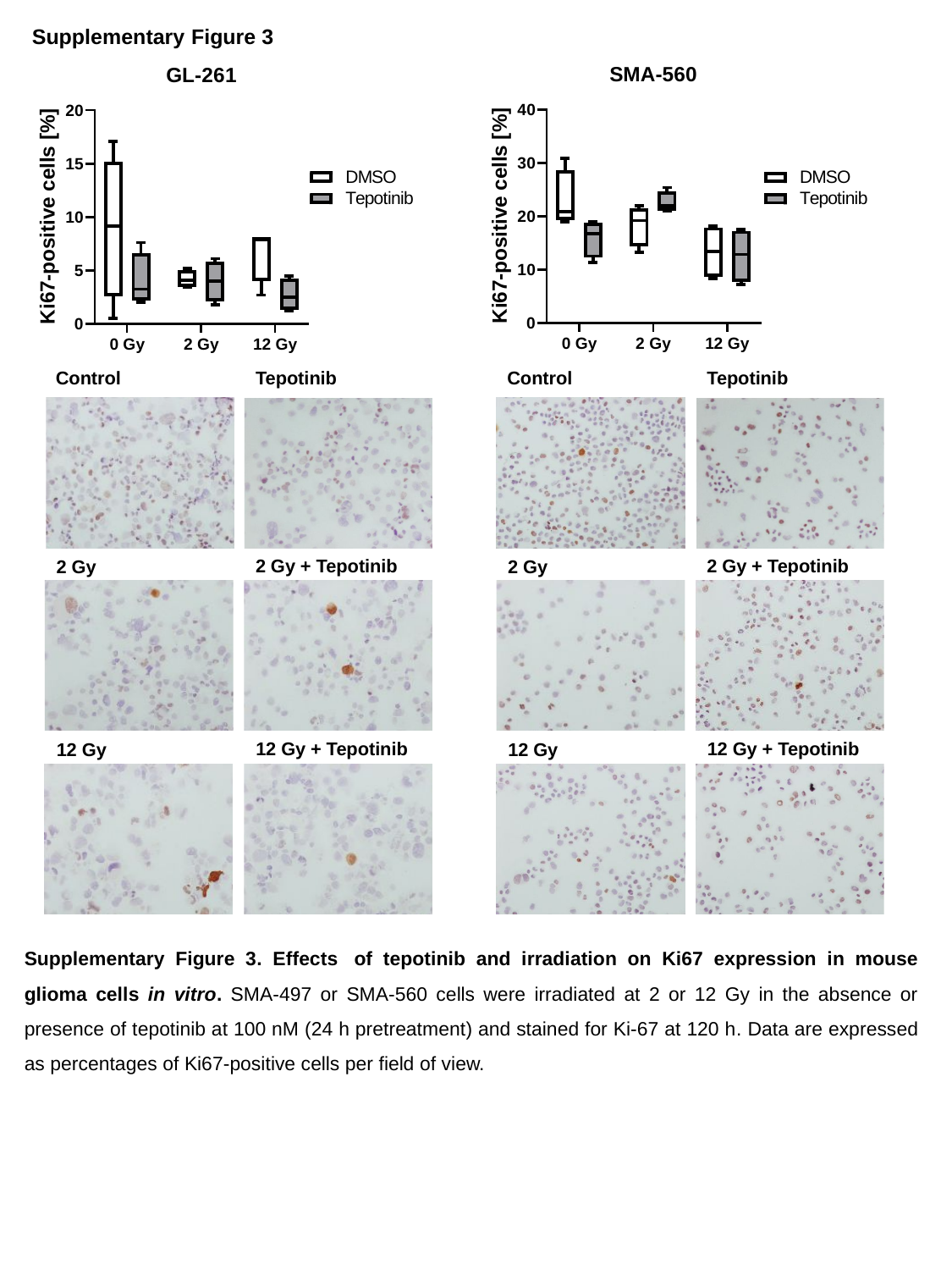

Supplementary Figure 3
Control
Tepotinib
Control
Tepotinib
2 Gy + Tepotinib
2 Gy + Tepotinib
2 Gy
2 Gy
12 Gy + Tepotinib
12 Gy + Tepotinib
12 Gy
12 Gy
Supplementary Figure 3. Effects  of tepotinib and irradiation on Ki67 expression in mouse glioma cells in vitro. SMA-497 or SMA-560 cells were irradiated at 2 or 12 Gy in the absence or presence of tepotinib at 100 nM (24 h pretreatment) and stained for Ki-67 at 120 h. Data are expressed as percentages of Ki67-positive cells per field of view.

## Slide 5
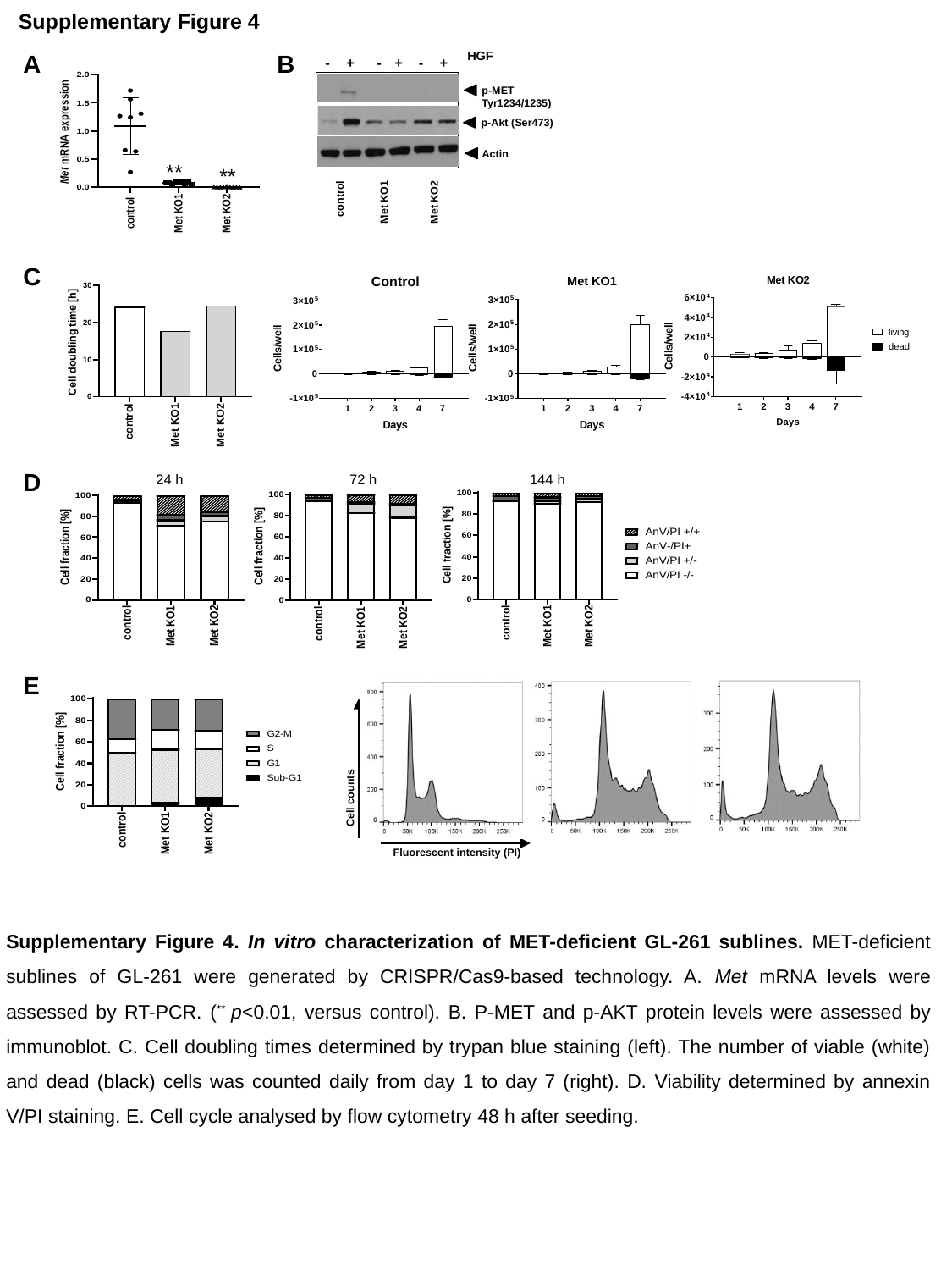

Supplementary Figure 4
A
B
HGF
-
+
-
+
-
+
p-MET Tyr1234/1235)
p-Akt (Ser473)
Actin
 control
 Met KO1
 Met KO2
**
**
C
D
24 h
72 h
144 h
E
Cell counts
Fluorescent intensity (PI)
Supplementary Figure 4. In vitro characterization of MET-deficient GL-261 sublines. MET-deficient sublines of GL-261 were generated by CRISPR/Cas9-based technology. A. Met mRNA levels were assessed by RT-PCR. (** p<0.01, versus control). B. P-MET and p-AKT protein levels were assessed by immunoblot. C. Cell doubling times determined by trypan blue staining (left). The number of viable (white) and dead (black) cells was counted daily from day 1 to day 7 (right). D. Viability determined by annexin V/PI staining. E. Cell cycle analysed by flow cytometry 48 h after seeding.

## Slide 6
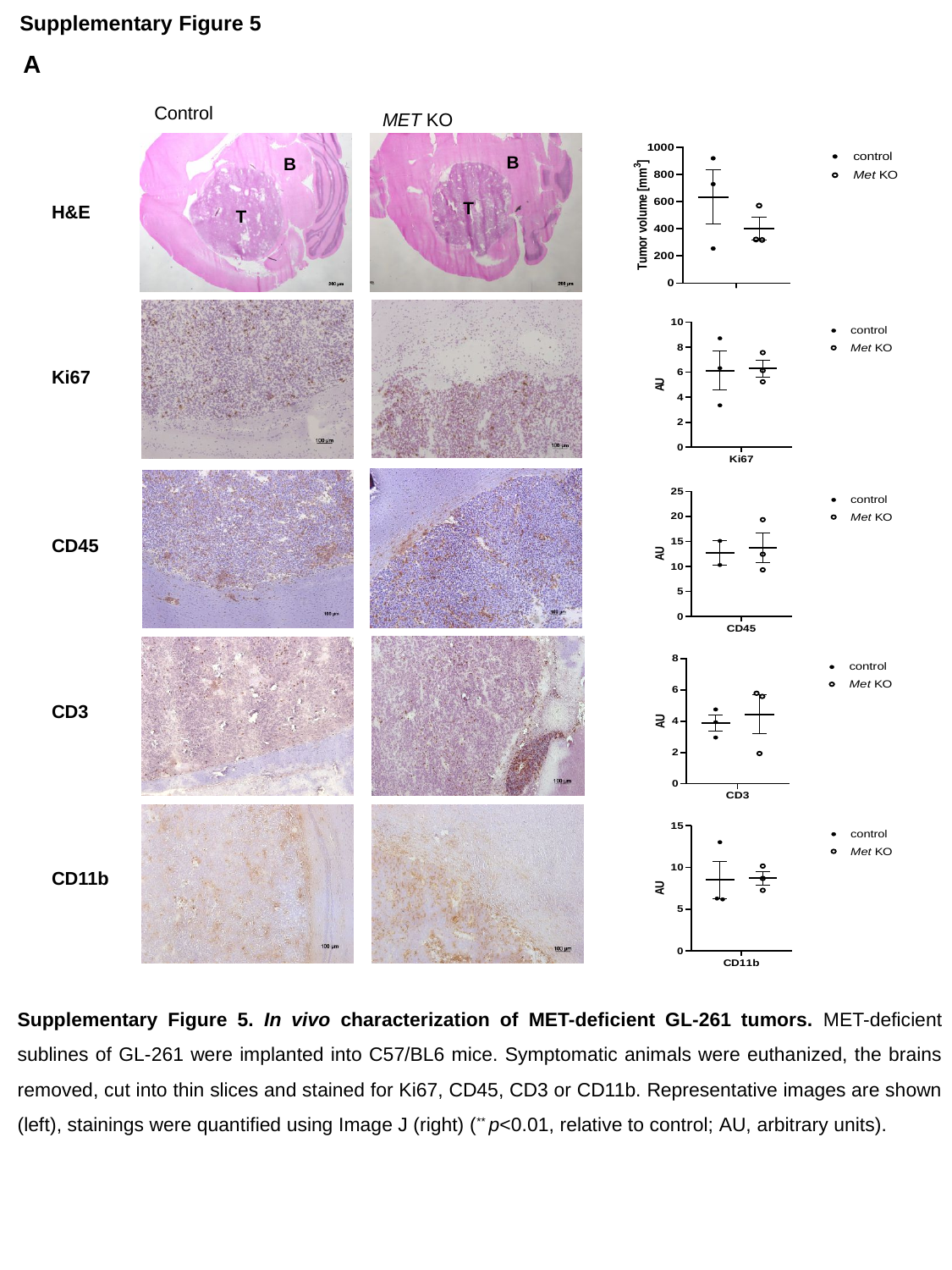

Supplementary Figure 5
A
Control
MET KO
B
B
T
H&E
T
Ki67
CD45
CD3
CD11b
Supplementary Figure 5. In vivo characterization of MET-deficient GL-261 tumors. MET-deficient sublines of GL-261 were implanted into C57/BL6 mice. Symptomatic animals were euthanized, the brains removed, cut into thin slices and stained for Ki67, CD45, CD3 or CD11b. Representative images are shown (left), stainings were quantified using Image J (right) (** p<0.01, relative to control; AU, arbitrary units).

## Slide 7
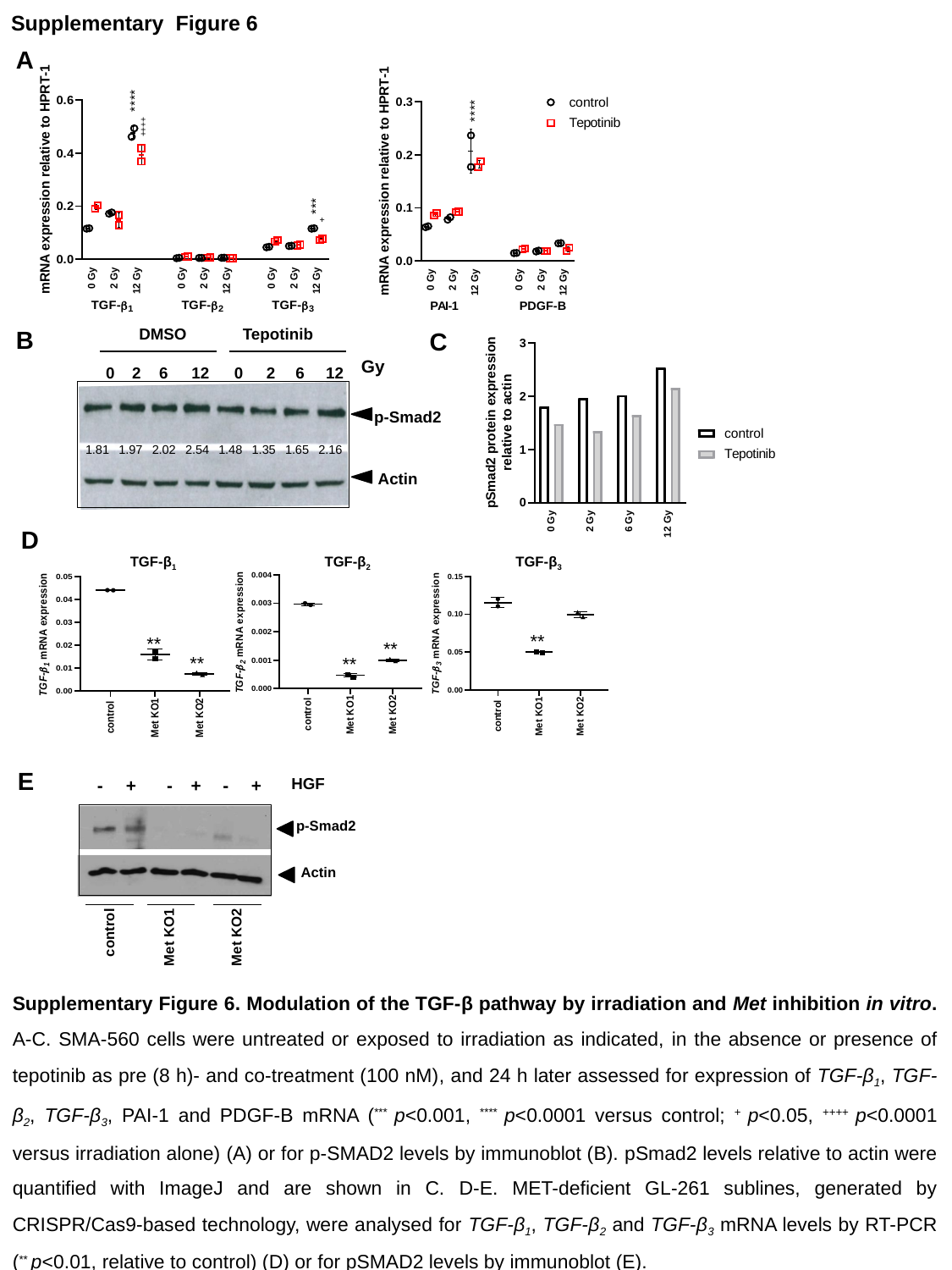

Supplementary Figure 6
A
Tepotinib
DMSO
Gy
0
2
6
12
0
2
6
12
p-Smad2
 1.81
 1.97
 2.02
 2.54
 1.48
 1.35
 1.65
 2.16
Actin
B
C
D
 TGF-β2
 TGF-β3
 TGF-β1
**
**
**
**
**
E
HGF
-
+
-
+
-
+
p-Smad2
Actin
 control
 Met KO1
 Met KO2
Supplementary Figure 6. Modulation of the TGF-β pathway by irradiation and Met inhibition in vitro. A-C. SMA-560 cells were untreated or exposed to irradiation as indicated, in the absence or presence of tepotinib as pre (8 h)- and co-treatment (100 nM), and 24 h later assessed for expression of TGF-β1, TGF-β2, TGF-β3, PAI-1 and PDGF-B mRNA (*** p<0.001, **** p<0.0001 versus control; + p<0.05, ++++ p<0.0001 versus irradiation alone) (A) or for p-SMAD2 levels by immunoblot (B). pSmad2 levels relative to actin were quantified with ImageJ and are shown in C. D-E. MET-deficient GL-261 sublines, generated by CRISPR/Cas9-based technology, were analysed for TGF-β1, TGF-β2 and TGF-β3 mRNA levels by RT-PCR (** p<0.01, relative to control) (D) or for pSMAD2 levels by immunoblot (E).
